# Quantification of polyoocyte follicles (POFs) in domestic cats

**DOI:** 10.1590/1984-3143-AR2024-0127

**Published:** 2025-07-04

**Authors:** Alexandra Lays Petri, Mariana Valentini Casagrande, Adalgiza Pinto, Jonatas Cattelam, Camila Keterine Gorzelanski Trenkel, Jacqueline Zanella, Leonardo Gruchouskei

**Affiliations:** 1 Laboratório de Reprodução Animal – LABRA, Universidade Federal da Fronteira Sul – UFFS, Realeza, PR, Brasil; 2 Universidade Federal de Santa Catarina – UFSC, Florianópolis, SC, Brasil

**Keywords:** feline, ovary, folliculogenesis, oocyte

## Abstract

Ovarian follicles usually involve only one oocyte. However, the existence of follicles with more than one oocyte has been described in different species. Therefore, this study evaluated the occurrence and number of Polyoocyte Follicles (POFs) in the ovaries of cats undergoing the ovariohysterectomy technique. For this purpose, ovaries from 33 cats were collected and submitted to histological preparation and analysis. The slides were evaluated under a microscope by two evaluators, who quantified the presence of follicles containing one or more oocytes and classified the follicular stage. Monoocyte follicles (MOFs) were found in all ovaries. POFs were observed at similar frequencies in both the right and left ovaries. The frequency of POFs with two oocytes was 79.03% in the right ovaries and 75.75% in the left ovaries. Follicles with three oocytes were found in 16.12% of the right ovaries and 27.27% of the left ovaries. The mean number of follicles with zero, one, two, three or more oocytes was similar between the ovaries, being 6.58, 310.5, 3.25, and 0.29 in the right ovary and 6.25, 312.7, 4.25, and 0.41 in the left ovary (p-values ​​of 0.7569, 0.9654, 0.4785, and 0.5015, respectively). POFs containing from two to five oocytes were identified in different stages of development. POFs at all stages of development were found in both ovaries, and the occurrence of these structures was influenced by the age, number of pregnancies, number of estrus, and weight of the cats studied.

## Introduction

Follicles are the functional units of the ovaries and can be classified according to their histological structure as primordial, primary, secondary, tertiary, and preovulatory follicles. The main function of these structures is to house and actively participate in the development of oocytes (Hafez and Hafez, 2004).

In domestic species, each ovarian follicle usually contains only one oocyte inside. However, the presence of follicles with more than one oocyte, called Polyoocyte Follicles (POFs), has been observed mainly in female dogs, cats, rabbits, opossums, and monkeys (Hartman, 1926; Telfer and Gosden, 1987; Bristol-Gould and Woodruff, 2006). The origin, development, viability, and correlation with fertility of POFs are still issues that science has not fully clarified. Even so, there are descriptions of the possibility of oocytes from these follicles developing *in vitro* until the blastocyst stage (Silva-Santos and Seneda, 2011).

Furthermore, it was found that POFs are more frequent in bitches at younger ages and decrease numerically as they mature (Payan-Carreira and Pires, 2008). Factors such as the use of contraceptives, pregnancy, and the animals' size have been shown to influence the presence of POFs (Zoppei et al., 2021).

Considering the scarcity of studies, this study aimed to verify the occurrence and frequency of POFs in the ovaries of cats undergoing the ovariohysterectomy technique, correlating them with the number of estrus, number of pregnancies, age, and weight of the animals studied.

## Methods

The present study was released from the Ethics Committee procedures for the use of animals, as the researchers did not have direct contact with the animals studied and the ovarian samples used were donations from the institutions that were partners in this research. The ovaries were collected from 33 domestic cats (*Felis catus*) undergoing ovariohysterectomy in veterinary clinics and hospitals that were partners in this study located in the municipalities of Pato Branco and Realeza (Paraná) after the animal’s owners signed the Free and Informed Consent Form. Data on the age, breed, weight, number of estrus, and pregnancies of the females were also collected.

Sample histological preparation was performed according to the proposal by Junqueira and Carneiro (2013). The samples were fixed with 4% formaldehyde, dehydrated by different concentrations of ethanol (in consecutive dilutions of 70, 80, 90, and 100%) and xylene (two one-hour baths), embedded in paraffin (two one-hour baths), and stained with hematoxylin and eosin. Histological slides were then made with a microtome at a thickness of 4 µm.

Just one histological slide of each ovary was examined in its entirety using an optical microscope (Olympus CX 33R), with objective lenses with magnifications of 40 to 400 times to evaluate the follicular population.

The ovarian follicular population was classified into monoocyte follicles (MOFs) when they had a single oocyte inside and polyoocyte follicles (POFs) when they had two or more oocytes. The number of oocytes in each POF was also collected. Morphologically, all follicles were classified according to Bristol-Gould and Woodruff (2006) as primordial, primary, secondary, and tertiary or Graafian follicles.

Follicles were classified as primordial when surrounded by a layer of granulosa cells, as primary when surrounded by a layer of cuboidal cells, as secondary when there were at least two layers of granulosa cells and one layer of theca cells, and as tertiary when there was the presence of the follicular antrum and two or three layers of theca cells. When examining histological slides obtained from very proximal or distal areas in relation to the corona radiata, it is possible that some tertiary follicles may not have visible oocytes. As a result, these follicles would be classified as tertiary follicles without oocytes.

Each histological slide was analyzed by two evaluators, considering the mean value between the readings. Data were tabulated in spreadsheets using Microsoft Excel and subjected to analysis of variance using the F test and analysis of variance using PROC GLM. The means were classified by the F test and the parameters with significant effect were compared by the “t” test with a significance level of p<0.05. The occurrence frequencies of MOFs and POFs were compared by the frequency dispersion test (chi-square) with a significance level of p<0.05. The analyses were performed using the SAS 9.2 statistical package. The ovaries and collected data analyses were carried out at the Animal Reproduction Laboratory of the Federal University of Fronteira Sul.

## Results

**Figure 1:**
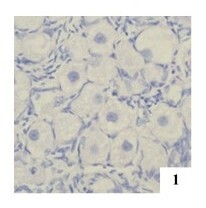
Primordial monoocyte follicle (MOF) in the right ovary of an adult female cat, H&E 400x.

**Figure 2:**
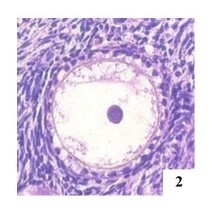
Primary MOF in the left ovary of an adult female cat, H&E 400x.

**Figure 3:**
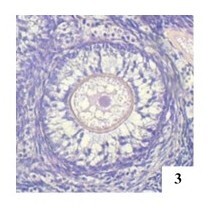
Secondary monoocyte follicle MOF in the left ovary of an adult female cat, H&E 400x.

**Figure 4:**
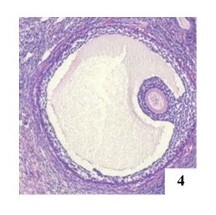
Tertiary MOF in the left ovary of an adult female cat, H&E 40x.

**Figure 5:**
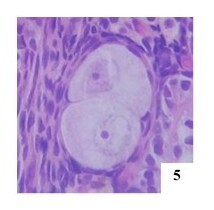
Primordial polyoocyte follicle (POF) with two oocytes in the right ovary of an adult female cat, H&E 400x.

**Figure 6:**
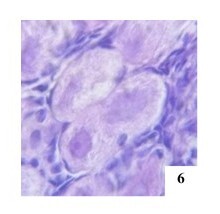
Primordial POF with three oocytes in the left ovary of an adult female cat, H&E 400x.

**Figure 7:**
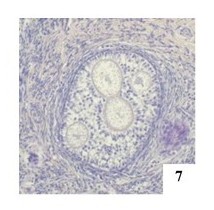
Secondary POF with four oocytes in the left ovary of an adult female cat, H&E 100x.

In all ovaries analyzed, MOFs were observed at different stages of development (Figures 1 to 4). The frequency and the average number of POFs containing two, three, and four oocytes (Figures 5 to 7) were similar in the right and left ovaries (p>0.05 - Table 1).

**Table 1:** Frequency and the average number of monoocyte follicles (MOFs) containing one oocyte and polyoocyte follicles (POFs) containing two, three, or four oocytes in the right (RO) and left (LO) ovaries of adult female cats.

**Ovary**	**Frequency (%) and average number of mono and polyoocyte follicles (%)**							
	**Oocytes per follicle**							
	**Zero**		**One**		**Two**		**Three or more**	
**RO**	-	6.58	100.00%	310.5	79.03%	3.25	19.34%	0.29
**LO**	-	6.25	100.00%	312.7	75.75%	4.25	31.81%	0.41
**p-value**		0.7569		0.9654		0.4785		0.5015

**Figure 8:**
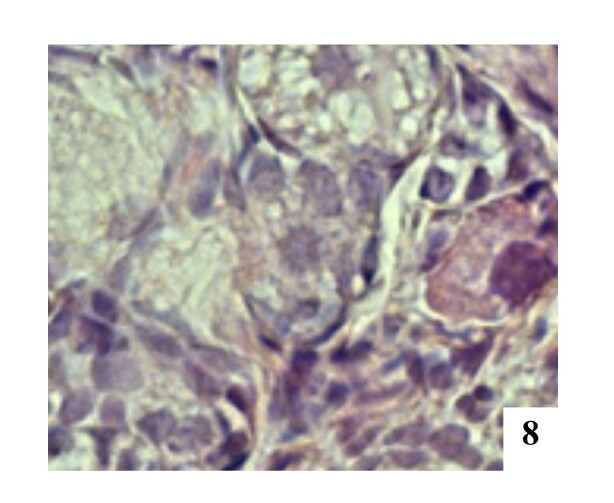
POF presenting five oocytes in the left ovary, H&E 400x.

In the present study, the frequency of MOFs in ovaries was higher than POFs (Table 1). The frequency and the average number of POFs decreased as the number of oocytes increased (Table 1), with only one POF containing five oocytes being observed (Figure 8).

Two evaluators obtained a mean of 22,816 follicles from the histological evaluation of ovarian sections, with 12,033 follicles identified and classified in the right ovaries and 10,784 in the left ovaries. Of these, 221 were classified as POFs (109 in the right ovaries and 112 in the left ovaries). Thus, when analyzing the total number of follicles found, it was observed that POFs constitute approximately 0.97% of the follicular population of the ovaries, regardless of the classification or ovary, in all histological sections analyzed.

Considering the 22,816 follicles quantified in the cats’ ovaries, 20,696 were classified as primordial (10,819 in the right ovary and 9,877 in the left ovary), 1,135 as primary (636 in the right ovary and 499 in the left ovary), 368 as secondary (226 in the right ovary and 143 in the left ovary) and 617 as tertiary follicles (352 in the right ovary and 265 in the left ovary).

Considering the small population, it was not possible to establish a difference in the number of POFs within each follicular classification. However, a variation in the frequency of their occurrence in the right and left ovaries was observed (Table 2).

**Table 2:** Frequency of monoocyte follicles (MOFs) and polyoocyte follicles (POFs) in the right (RO) and left (LO) ovaries of adult cats.

**Follicular classification**	**Ovary**	**Number of oocytes**					
		**Zero**	**One**	**Two**	**Three**	**Four**	**Five**
**Primordial Follicles (%)**	**RO**	0.00	100.00	72.58	14.51	0.00	0.00
	**LO**	0.00	100.00	71.21	18.18	4.54	1.51
**Primary Follicles (%)**	**RO**	0.00	96.77	22.58^a^	1.61	0.00	0.00
	**LO**	0.00	96.96	9.09^b^	1.51	0.00	0.00
**Secondary Follicles (%)**	**RO**	0.00	88.70	11.29^a^	0.00	3.22	0.00
	**LO**	0.00	93.93	3.03^b^	1.51	0.00	0.00
**Tertiary Follicles (%)**	**RO**	95.16	88.70	3.22^b^	0.00	0.00	0.00
	**LO**	95.45	83.33	13.63^a^	0.00	0.00	0.00

^ab^Values ​​followed by different letters in the same column for the same follicular classification are significantly different (p<0.05).

Oocytes at different stages of development were observed in POFs, distinguished by the zona pellucida thickness (Figure 7). Primordial, primary, secondary and tertiary POFs were observed in the ovaries at average frequencies of 95.44%, 17.40%, 9.53% and 8.43%, respectively. The classification of POFs by the number of oocytes in the right and left ovaries is shown in Table 2. POFs with one, two, three, four and up to five oocytes were observed in primordial, primary and secondary follicles. In tertiary follicles, POFs with two oocytes were observed.

All ovaries studied presented primordial, primary, secondary, and tertiary follicles (Table 2).

**Figure 9:**
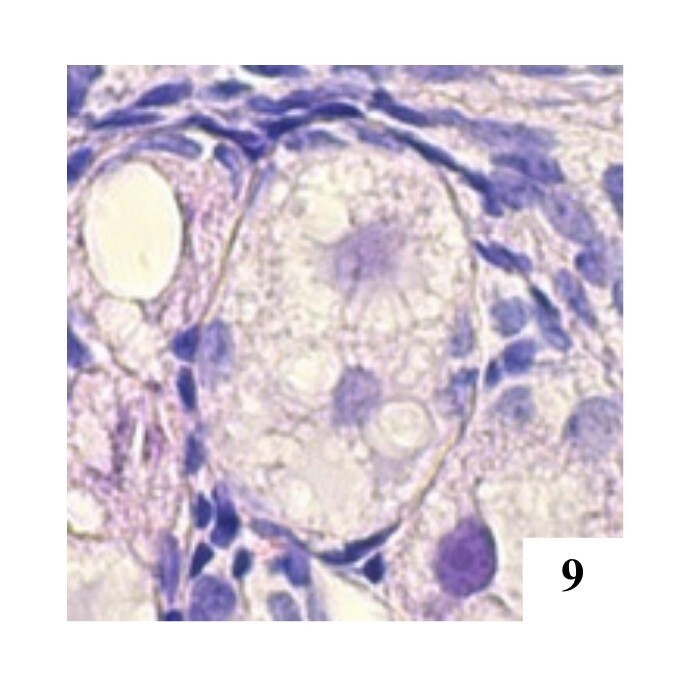
Primordial POF with two oocytes in the left ovary, H&E 400x.

**Figure 10:**
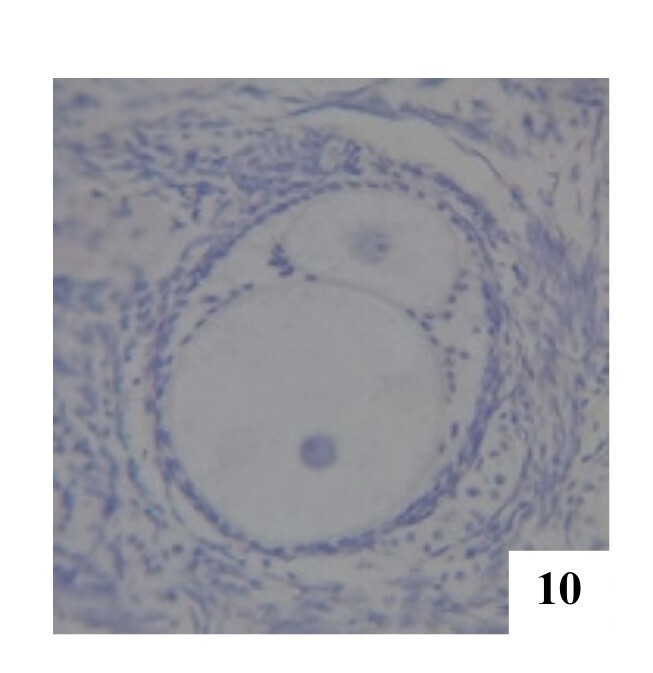
Primary POF with two oocytes in the left ovary, H&E 100x.

**Figure 11:**
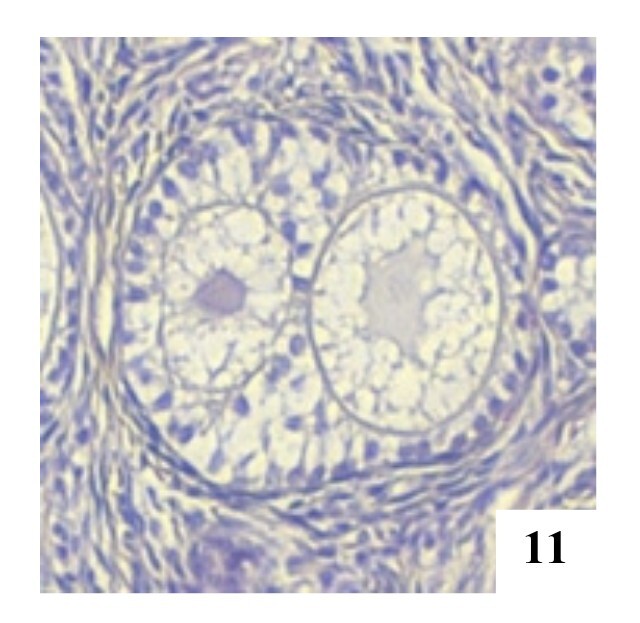
Secondary POF with two oocytes in the right ovary, H&E 100x.

**Figure 12:**
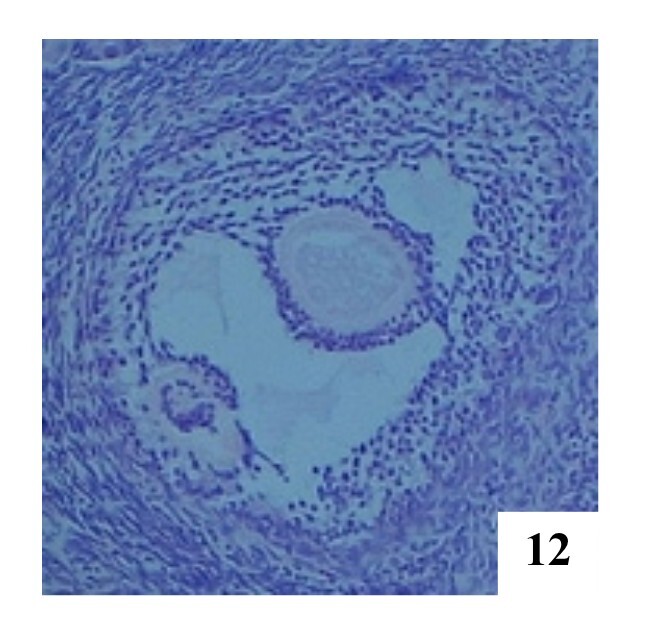
Tertiary POF with two oocytes in the left ovary, H&E, 40x.

In this study, POFs were found in all developmental stages (Table 3 and Figures 9 to 12).

**Table 3:** Average number of monoocyte follicles (MOFs) and polyoocyte follicles (POFs) in the ovaries of adult cats according to age.

**Age of cats**	**Average number of MOFs and POFs**			
	**Number of oocytes**			
	**Zero**	**One**	**Two**	**Three or more**
**Up to 12 months**	5.72	372.1^a^	4.04	0.27
**12-24 months**	7.21	376.7^a^	2.67	0.17
**24-48 months**	7.93	298.2^ab^	2.37	0.37
**More than 48 months**	5.70	218.4^b^	1.50	1.15
p-value	0.1830	0.0045	0.0656	0.5893

^ab^Values ​​followed by different letters in the same column are significantly different (p<0.05)

The mean number of MOFs reduced as animals age (p<0.05), and this reduction was observed in cats older than 48 months (Table 3).

The mean number of POFs containing two, three, or more oocytes was similar as cats were aging (p>0.05 - Table 4).

**Table 4:** Average number of monoocyte follicles (MOFs) and polyoocyte follicles (POFs) in the ovaries of adult cats according to the number of pregnancies.

**Number of pregnancies**	**Average number of MOFs and POFs**			
	**Number of oocytes**			
	**Zero**	**One**	**Two**	**Three or more**
**None**	7.00	282.7^b^	3.25	0.10^b^
**One**	5.85	281.0^b^	3.15	0.25^b^
**Two or more**	6.37	460.5^a^	6.50	1.25^a^
p-value	0.6218	0.0255	0.2125	

^ab^Values ​​followed by different letters in the same column are significantly different (p<0.05)

In the present study, the average number of MOFs was higher (p<0.05) in cats with two or more pregnancies than in nulliparous cats or those with only one pregnancy. The average number of POFs with two oocytes was similar between nulliparous cats and those with at least one pregnancy (p>0.05). Nevertheless, cats with two or more pregnancies had a higher average number of POFs with three or more oocytes (p<0.05), as observed in Table 4.

Moreover, pre-pubertal cats had a higher average number of MOFs than pubescent cats (p<0.05 - Table 5).

**Table 5:** Average number of monoocyte follicles (MOFs) and polyoocyte follicles (POFs) in the ovaries of adult cats according to the number of estrus.

**Number of estrus**	**Average number of MOFs and POFs**			
	**Number of oocytes**			
	**Zero**	**One**	**Two**	**Three or more**
**None**	8.50	512.7^a^	3.50	0.00^b^
**One**	5.40	261.7^b^	3.67	0.21^a^
**Two or more**	7.68	348.7^ab^	3.93	0.68^a^
**p-value**	0.648	0.0118	0.9805	0.0265

^ab^Values ​​followed by different letters in the same column are significantly different (p<0.05)

The mean number of POFs with two oocytes was similar in pre-pubertal and pubertal cats (p>0.05). However, cats that presented two or more estrus had more POFs with three or more oocytes (p<0.05 - Table 5).

In this study, cats' weight influenced the presence of MOFs and POFs (Table 6). A higher mean number of MOFs and POFs with two oocytes was observed in the ovaries of cats weighing less than 2 kg and more than 3.1 kg (p<0.05). However, cats weighing over 3.1 kg had more POFs with three or more oocytes (p<0.05).

**Table 6:** Average number of monoocyte follicles (MOFs) and polyoocyte follicles (POFs) in the ovaries of adult cats according to adult weight.

**Weight**	**Average number of MOFs and POFs**			
	**Number of oocytes**			
	**Zero**	**One**	**Two**	**Three or more**
**Up to 2 Kg**	7.66	398.2^a^	6.16^a^	0.08^b^
**2.1-3.0 Kg**	6.37	247.1^b^	1.79^b^	0.16^b^
**3.1 Kg or more**	5.25	354.1^ab^	5.25^a^	1.00^a^
**p-value**	0.272	0.0260	0.0139	<0.0001

^ab^Values ​​followed by different letters in the same column are significantly different (p<0.05).

## Discussion

The decline in the frequency of POFs as the number of oocytes increased (Table 1), corroborating reports described in studies with women, sows, and bitches (Gougeon, 1981; Stankiewicz et al., 2009; Payan-Carreira and Pires, 2008). Furthermore, POFs containing only two oocytes are more commonly observed than those containing three or more oocytes (Telfer and Gosden, 1987; Shehata, 1974; Cardoso, 2017).

The difference in the frequency of MOFs and POFs in the right and left ovaries (Table 1) found in this study coincides with that described by Zoppei et al. (2021), who did not observe differences between the follicular population when studying the ovaries of 38 bitches. However, Lunardon et al. (2015), when reporting a difference between the number of follicles in the left and right ovaries, added that the observation of only one histological section per ovary may overestimate or underestimate the follicular population.

The highest frequency of MOFs in cats’ ovaries (Table 1) coincides with the reports of Telfer and Gosden (1987) when they studied the ovaries of fifteen different species of mammals. Shehata (1974) also observed sporadic presence of POFs in feline ovaries and smaller numbers than MOFs.

The frequency of POFs decreased as the number of oocytes increased (Table 1). The number of COCs (cumulus oophorus complexes) observed in POFs varies according to the species analyzed (Silva-Santos and Seneda, 2011). However, Hartman (1926) reported the POF with the highest number of oocytes in felines described in the literature. The author observed eight oocytes in a follicle when evaluating a feline female's ovary. Other authors, such as Dederer (1934), verified the existence of five and six oocytes in different POFs when evaluating 388 slides with different histological sections of the ovaries of an approximately one-year-old cat.

The mean number of MOFs and POFs similar between the ovaries of all animals studied (p>0.05 - Table 1) was similar to reports described by Zoppei et al. (2021) when evaluating the ovaries of bitches. The authors observed that the occurrence of MOFs and POFs with four or more COCs in both ovaries was similar. Payan-Carreira and Pires (2008) also reported the bilateral occurrence of MOFs in the ovaries of both pubertal and pre-pubertal bitches, presenting different occurrences for these categories.

Oocytes at different stages of development in POFs (Figure 7) were similar findings described the presence of oocytes within the same POF varying in size or being degenerated, which suggests that these oocytes were at different stages of development. Other studies also observed oocytes of different sizes and degenerated in the same follicle (Miclăuş et al., 2007).

Shehata (1974) also observed atresia in one of the POF oocytes when it contained two oocytes. Silva-Santos and Seneda (2011) reported that when there are two oocytes within a follicle, they are in the same state of development. Still, this synchrony is not observed in follicles with three or more oocytes since the smallest oocyte can trigger oocyte degeneration.

On the other hand, in a study using ovaries from cats and dogs, Cardoso (2017) found that oocytes present in the same follicle demonstrated a similar pattern in the expression of Proliferating Cell Nuclear Antigen (PCNA), a marker of cell proliferation, suggesting that they were in the same stage of development. Oliveira et al. (2017), when analyzing the ovaries of a Santa Ines sheep, proposed that oocytes from the same follicle may be in the same stage of development after histological investigation and demonstrate similar morphological appearance and staining patterns when using the PCNA technique. In addition, previous authors had already reported follicles with oocytes in similar stages of development (Hartman, 1926; Shehata, 1974).

All ovaries studied presented at different development stages follicles (Table 2). Carrijo et al. (2010) also found a diverse follicular population, reporting 87% primordial, 10.4% primary, and 2.3% secondary follicles in panda ovaries. Previous studies have shown that primordial and primary follicles may represent 80-90% of the total population in cats, while secondary follicles represent 10-20% (Jewgenow and Goritz, 1995).

POFs were found in all developmental stages (Table 2 and Figures 9 to 12). Authors such as Hartman (1926), Dederer (1934), Shehata (1974), and Miclăuş et al. (2007) reported similar results. However, Uchikura et al. (2010) and Cardoso (2017) observed POFs in the secondary, preantral, and antral stages. Monteiro et al. (2006) observed only preantral and antral POFs.

The MOFs reduced as animals age (p<0.05). Miclăuş et al. (2007), when analyzing the ovaries of two female cats, revealed that the reserve of primordial follicles was significantly lower in the four-year-old animal in relation to the ovaries of the seven-month-old animal. However, the older cat's ovary had follicles at different evolutionary stages.

The POFs containing two, three, or more oocytes were similar as cats were aging (p>0.05 - Table 3). However, a statistical trend (p=0.06) of a higher mean number of POFs with two oocytes was observed in cats up to 12 months old. Shehata (1974) observed the presence of POFs in the ovaries of a newborn cat but not in the ovaries of adolescent or adult cats. Furthermore, Cardoso (2017) evaluated the ovaries of 11 cats and showed a higher occurrence of POFs in young animals, while Uchikura et al. (2010) found a similar percentage of POFs in cats less than 20 days old and up to 120 days old.

In bitches, the prevalence of POFs was higher in pre-pubertal animals (68%), decreasing to 62% in bitches under one year old. This percentage dropped by half when the animals reached eight years of age and reached 14% in purebreds when they reached more than ten years (Payan-Carreira and Pires, 2008). Zoppei et al. (2021), studying bitch ovaries, reported that the animal's age does not alter the presence of POFs.

It is known that folliculogenesis in domestic cats begins around three weeks of fetal life and that, during oogenesis, mitotic multiplications of the oocyte occur until approximately eight days after birth (Bristol-Gould and Woodruff, 2006; Cardoso, 2017). However, the number of oocyte predetermination in mammals or the existence of stem cells that can differentiate during adult life are hypotheses under investigation and not yet fully clarified (Johnson et al., 2004).

Thus, the decrease in the average number of oocytes, whether in MOFs or POFs, with advancing age may corroborate the hypothesis of the predetermined number of ovarian follicles. Cats already use the induced ovulation mechanism to prevent wasting follicles, so they would not need stem cells to differentiate into germinal structures after birth. Therefore, their reserve would be used during their reproductive life by ovulation or atresia of these structures, with no follicular replacement and leading to a decrease in the follicular population as age advances (Bristol-Gould and Woodruff, 2006).

In the present study, the average number of MOFs and POFs varied with the number of pregnancies (Table 4). Monteiro et al. (2006), when histologically analyzing the ovaries of nulliparous and multiparous cats, observed two ovaries that presented pre-antral and antral POFs in the first group of animals. In the second group, only one ovary had follicles with more than one oocyte. They concluded that multiparous females have a higher mean number of POFs, which was also observed in this study.

Findings different from those observed in the present study were described by Zoppei et al. (2021). The authors analyzed the ovaries of 38 bitches and reported that the number of pregnancies influenced the frequency of POFs. However, follicles that presented up to three COCs were more frequent in the ovaries of nulliparous bitches, and follicles with four or more COCs were more frequent in the ovaries of bitches with only one pregnancy.

Pre-pubertal cats had a higher mean number of MOFs than pubescent cats, which had one, two, or more estrus (p<0.05 - Table 5). This result can be explained by the feline’s follicular dynamics that present follicular growth close to estrus, selecting the population with ovulatory potential and causing atresia of the others (Cardoso, 2017). Thus, the process of follicular atresia that occurs in each estrous cycle leads to a decrease in the ovarian follicular population (Araújo et al., 2021). Therefore, as observed in this study, older cats that have gone through more estrus have a smaller follicular population and a smaller population of MOFs.

The POFs with two oocytes were similar in pre-pubertal and pubertal cats (p>0.05). (p<0.05 - Table 5). This result could support the hypothesis concerning the influence of estrogen on the appearance of POFs, considering that cats that presented more estrus had greater exposure to this steroid. Mice exposed to genistein in the neonatal period presented greater induction of POF formation, as genistein stimulates the expression of estrogen receptors. Furthermore, the incidence of POFs and the number of oocytes per follicle in the ovaries of these mice correlated with the dose of genistein applied, with higher doses leading to a greater effect (Jefferson et al., 2002).

However, considering that an older animal has gone through more estrous cycles, it is reasonable to assume that the number of POFs would be lower in this animal. Age influences the mean number of POFs decrease, as they are ovulated or undergo atresia during life, and the existence of stem cells that differentiate into oocytes during adulthood, which would increase the number of these structures in the feline ovary, is not proven as previously mentioned.

In this study, cats' weight influenced the presence of MOFs and POFs (Table 6). No cat included in this study was overweight; this fact, associated with the slight variation in animals’ weight, could explain the difficulty in interpreting the results regarding the real influence of this variable on ovarian dynamics.

Astudillo et al. (2023), studying bitches, stated that oocytes from follicles with more than one oocyte can return to meiosis as much as MOFs, even though at slower rates, making in vitro maturation possible. Furthermore, these authors affirmed that the existence of POFs influences the oocyte recovery rate. Although this study did not aim to explain the origin of POFs and/or their influence on the fertility of cats, it is clear that the existence of POFs in cats’ ovaries is relevant to the study of reproductive biotechnologies. Thus, additional studies are necessary regarding these structures and their importance for the feline species, their ovulation capacity, and their fertilization potential, therefore contributing to the development of reproductive technologies in felines and the conservation of endangered feline species, for example.

## Conclusion

Under the conditions of this study, we concluded that the cats' ovaries presented POFs in all stages of follicular development, which constitutes 0.97% of the ovarian population, making it impossible to establish the number of POFs within each follicular classification. Follicles containing up to five oocytes were observed in primordial, primary and secondary follicles. Up to two oocytes were observed in tertiary follicles. No difference in frequency between the left and right ovaries was observed. Additionally, the oocytes present in the same POF may be in different stages of development, and the occurrence of these structures may be influenced by the age, number of pregnancies, number of estrus, and weight of the animals.

It is necessary to emphasize the scarcity of available literature on POFs in cats capable of supporting an in-depth discussion regarding the findings of this article, which, therefore, limits this study to reporting the initial findings on the follicular structures studied.
